# Crosstalk between liver macrophages and gut microbiota: An important component of inflammation-associated liver diseases

**DOI:** 10.3389/fcell.2022.1070208

**Published:** 2022-11-22

**Authors:** Ziyuan Zhou, Xiaxia Pan, Lanjuan Li

**Affiliations:** State Key Laboratory for Diagnosis and Treatment of Infectious Diseases, National Clinical Research Center for Infectious Diseases, Collaborative Innovation Center for Diagnosis and Treatment of Infectious Diseases, The First Affiliated Hospital, Zhejiang University School of Medicine, Hangzhou, China

**Keywords:** macrophage, gut microbiota, inflammation, liver disease, innate immunity, gut-liver axis

## Abstract

Hepatic macrophages have been recognized as primary sensors and responders in liver inflammation. By processing host or exogenous biochemical signals, including microbial components and metabolites, through the gut-liver axis, hepatic macrophages can both trigger or regulate inflammatory responses. Crosstalk between hepatic macrophages and gut microbiota is an important component of liver inflammation and related liver diseases, such as acute liver injury (ALI), alcoholic liver disease (ALD), and nonalcoholic fatty liver disease (NAFLD). This review summarizes recent advances in knowledge related to the crosstalk between hepatic macrophages and gut microbiota, including the therapeutic potential of targeting hepatic macrophages as a component of gut microecology in inflammation-associated liver diseases.

## Introduction

Macrophages serve as crucial components in the innate immune system. As the primary cells involved in phagocytosis, macrophages are fully equipped with diverse pattern recognition receptors (PRRs), including Toll-like receptors (TLRs) and scavenger receptors (SRs), adding powerful lysosomal enzymes to engage, recognize, and eliminate pathogenic agents (Janeway and Medzhitov, 2002; Nagata, 2018). The functions and phenotypes of macrophages vary widely, depending on physiologic and/or pathologic conditions (Gordon and Taylor, 2005; Gordon et al., 2014). For example, macrophages can polarize into two subsets: proinflammatory classically activated macrophages (M1) and anti-inflammatory alternatively activated macrophages (M2) (Murray and Wynn, 2011; Locati et al., 2020).

Two main categories of macrophages are present in the liver: liver-resident Kupffer cells (KCs) and circulating monocyte-derived macrophages (MoMφs) (Varol et al., 2015; Dou et al., 2019). In general, KCs are responsible for sensing and processing biochemical signals, such as pathogen components, cell fragments, and endo/exogenous metabolites (Diehl et al., 2020; Li W. et al., 2022). They can trigger primary inflammation and secrete chemokines to recruit MoMφs for further inflammatory responses (Wen et al., 2021; Zhou J. et al., 2022). Hepatic macrophages can also regulate inflammatory responses when stimulated with anti-inflammatory factors. Hence, the signaling pathways associated with macrophage activity and heterogeneity induced by different agents play a vital role in inflammation-associated liver diseases (Dou et al., 2019; Wen et al., 2021; Li W. et al., 2022).

Studies over the last few decades have introduced the concept of the gut-liver axis, which emphasizes the importance of crosstalk between gut-derived microbial agents and hepatic cells such as macrophages (Wu and Tian, 2017; Tripathi et al., 2018; De Muynck et al., 2021). Hepatic macrophages can trigger liver inflammation when stimulated by gut-derived signals such as pathogen-associated molecular patterns (PAMPs) (Thevaranjan et al., 2017; Toubal et al., 2020; Zhang et al., 2021b). In contrast, many microbial factors, such as short-chain fatty acids (SCFAs), can suppress or regulate hepatic macrophage activation (Bao et al., 2020; Wang Z. et al., 2020; Xia et al., 2022). This gut microbiota-macrophage-liver inflammation axis is highly significant in inflammation-related liver conditions.

The present review focuses on the crosstalk between hepatic macrophages and gut microbiota, as well as the association between specific microbes and inflammation-related liver conditions. Targeting hepatic macrophages as well as gut microbiota may have therapeutic potential in the treatment of inflammatory liver diseases.

## The origin and characteristics of hepatic macrophages

Hepatic macrophages consist primarily of two subsets, resident KCs and circulating MoMφs, which differ in origin and characteristics. Generally, tissue-resident KCs are seeded into the fetal liver during embryogenesis. One hypothesis states that KCs originate from CSF1R^+^ erythromyeloid progenitors (EMPs) present in the fetal liver during embryogenesis (Gomez Perdiguero et al., 2015). These EMPs develop in the yolk sac on embryonic day 8.5 (E8.5), subsequently developing into monocytes after colonizing the fetal liver. KCs are thought to originate from fetal liver monocytes as early as E12.5 (Hoeffel et al., 2015). A second hypothesis states that KCs originate from circulating CD45^+^, Kit^−^, and Lin^−^ macrophage precursors (pMac) derived from EMPs, which colonize the fetal liver beginning on E9.5 and dependent on CX3CR1, giving rise to tissue-resident macrophages as early as E12.5 (Mass et al., 2016). Similar to other tissue-resident macrophages, KCs possess self-renewing ability and are mostly independent from bone marrow (BM)-derived progenitors (Schulz et al., 2012; Yona et al., 2013; Gomez Perdiguero et al., 2015). In an unsteady state, circulating monocytes can replenish KC populations (Hettinger et al., 2013; Scott et al., 2016).

Mature KCs share a series of common macrophage surface markers, such as CD11 and F4/80, whereas KCs in mice express the unique marker, C-type lectin domain family 4 member F (CLEC4F) (Krenkel and Tacke, 2017; Li W. et al., 2022). These KCs usually inhabit liver sinusoids, where they continuously engage with and process senescent cells and various gut-derived particles. Generally, KCs in the liver have three primary activities: clearance of pathogens and cells that have undergone apoptosis (Toth and Thomas, 1992; Shi et al., 1996; Horst et al., 2019; Deppermann et al., 2021); antigen presentation and the induction/regulation of inflammation (Liu et al., 2015; Yu et al., 2019; Zhang et al., 2021a; Ni et al., 2021; Li W. et al., 2022); and iron, bilirubin, and lipid metabolism (Scott and Guilliams, 2018; Diehl et al., 2020; Bleriot et al., 2021). KCs are essential for both maintaining liver homeostasis and responding to liver injury.

MoMφs originate from CX3CR1^+^, CD117^+^, and Lin^–^progenitors, also called common monocyte progenitors (cMoP), in bone marrow (Fogg et al., 2006; Hettinger et al., 2013). In addition to common macrophage markers, MoMφs express unique CX3CR1 and CCR2 markers. Mouse MoMφs can be differentiated into two subsets according to their levels of expression of Ly6C. Ly6C^+^ cells can be recruited by chemokines and infiltrate the liver, where they have proinflammatory activity, whereas many Ly6C^−^ cells act as scavengers and regulators of inflammation (Carlin et al., 2013; Morias et al., 2015; Krenkel et al., 2018; Ambade et al., 2019). Another subset of CX3CR1^+^ Ly6C^−^ resident macrophages has been identified in liver capsules as liver capsular macrophages (LCMs), which can directly take up both particulate antigens such as LPS and bacteria in capsule with extended dendrites. They are replenished by circulative monocytes and can recruit neutrophil infiltration (Sierro et al., 2017).

Classically, the observation of the macrophage polarization under interferon gamma (IFN-γ) or interleukin 4 (IL-4) stimulation identified the distinct biologic profile between pro-inflammatory M1 (mainly secreting TNF-α and IL-1β) and anti-inflammatory M2 macrophages (mainly secreting IL-10), respectively. (Murray and Wynn, 2011; Murray, 2017). With the increasing understanding in recent years, the role of inflammation or resolution in hepatic macrophages were found to be interwoven and rapidly transformed (Ramachandran et al., 2012; Beattie et al., 2016; Mossanen et al., 2016). Some scholars have suggested that a more specific and dynamic method to clarify polarization of hepatic macrophages is needed under complex pathologic conditions (Xue et al., 2014; Tacke, 2017; Locati et al., 2020). However, the evidence of microbial regulation on hepatic macrophage polarization has not gone that far yet. The classic concept of macrophages polarization indicates a general view of functional heterogeneity, which is still helpful to describe the characteristics of hepatic macrophages here.

## Hepatic macrophages in liver inflammation

As members of the innate immune system, hepatic macrophages are crucial initiators of and participants in liver inflammation. They are mainly responsible for triggering inflammatory responses to various insults but may also cause severe tissue injury and dysfunction (Koyama and Brenner, 2017; Tacke, 2017; Locati et al., 2020).

In general, liver inflammation is initiated by the engagement between KCs and innate molecular patterns (IMPs), including PAMPs and damage-associated molecular patterns (DAMPs) (Dou et al., 2019; Li W. et al., 2022). PRRs such as TLRs play an important role in recognizing DAMPs, including free DNA and high-mobility group box 1 (HMGB1) from damaged hepatocytes, and in recognizing PAMPs, such as lipopolysaccharides (LPS), lipoproteins, and peptidoglycan (PGN) (Takeda et al., 2003; Takeuchi and Akira, 2010; Kumar et al., 2011). Upon recognizing IMPs, KCs can secrete proinflammatory agents (e.g., IL-1β, CCL2) through inflammatory signaling pathways (e.g., NF-κB, PI3K/AKT) to recruit or activate downstream cells (e.g., MoMφs, T cells, and vascular endothelial cells) (Janeway and Medzhitov, 2002; Taylor et al., 2005; Wynn et al., 2013).

MoMφs usually take part in the second stage of liver inflammation. Ly6C^+^ MoMφs can be recruited by KC-secreted chemokines such as CCL2 and massively infiltrate into injured liver to replenish hepatic macrophages and participate in further inflammation. In many inflammation-related liver diseases, Ly6C^+^ MoMφs act as potent proinflammatory cells, secreting a large number of cytokines and inducing tissue injury (Krenkel et al., 2018; Ambade et al., 2019; Dai et al., 2020; Kolodziejczyk et al., 2020). Inhibition of CCL2/CCR2 signaling, which blocks the recruitment of Ly6C^+^ MoMφs, is a potential therapeutic target in liver inflammation (Krenkel et al., 2018; Ambade et al., 2019). In addition, a subset of resident Ly6C^−^ MoMφs is responsible for sensing pathogens and recruiting neutrophils (Sierro et al., 2017).

**FIGURE1:**
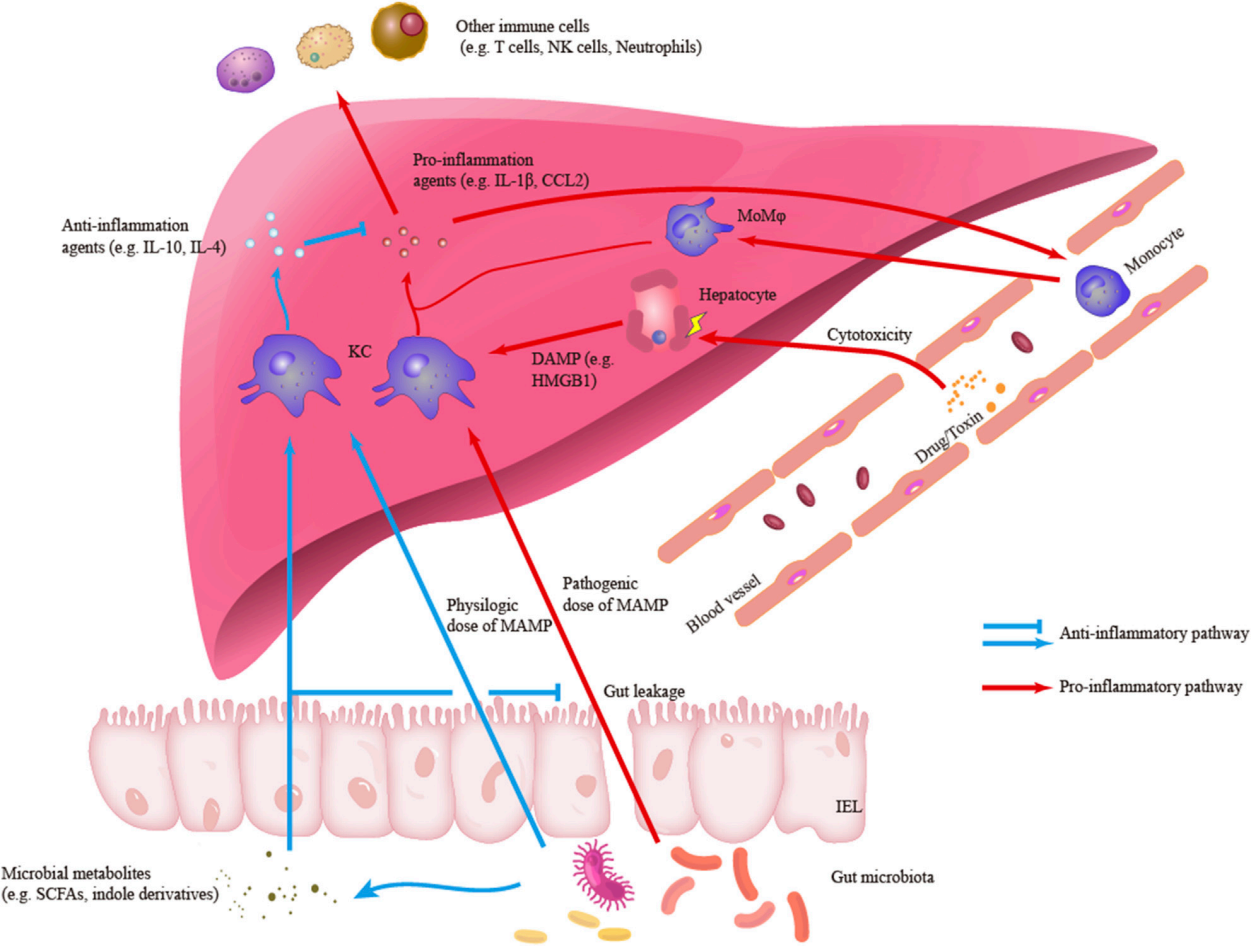
Outlines of the roles of hepatic macrophages in liver inflammation. Kupffer cells (KCs) can engage and response to different signals including pro-inflammatory damage-associated molecular patterns (DAMP) and microbe-associated molecular patterns (MAMP) or anti-inflammatory MAMP and microbial metabolites. In the pro-inflammatory response, KCs can secrete pro-inflammatory agents such as IL-1β and CCL2, which are responsible to initiate liver inflammation and recruit other immune cells to infiltrate liver. Another main subset of hepatic macrophage, monocyte-derived macrophages (MoMφs), is also an important component in the inflammatory infiltration. Besides, in the anti-inflammatory response, KCs can secrete anti-inflammatory agents and regulate inflammatory signaling and response.

Hepatic macrophages are also essential in the resolution of liver inflammation and the repair of liver injury. Reductions in the numbers of macrophages can decrease the proliferation of hepatocytes and the restoration of tissue homeostasis (Tacke, 2017). Hepatic macrophages with an anti-inflammatory phenotype, including Ly6C^−^ MoMφs, can promote the clearance of proinflammatory agents, such as cleaved IL-1β, and release cytokines with anti-inflammatory and wound-healing effects, such as IL-4 (Mosser and Edwards, 2008; Kim and Nair, 2019; Starkey Lewis et al., 2020). In addition, KCs are crucial components in the establishment of hepatic immune tolerance (Knolle et al., 1995; Chou et al., 2015; Doherty, 2016). KCs can both be activated or secret IL-10 to maintain an immunosuppressive environment in liver cancer and acute liver injury (ALI) (Erhardt et al., 2007; Wu et al., 2009). Meanwhile, KCs are capable to down-regulate own TLR4-signaling pathways under the long-term exposure to gut-derived LPS (low dose) partly due to the induction of interleukin-1 receptor-associated kinase M. This LPS tolerance may be necessary to maintain the homeostasis of the gut-liver axis (Liu et al., 2008). A recent study also identified that KCs treated with polymeric nanoparticles which contain disease-relevant antigens are sufficient to mediate immune tolerance in experimental autoimmune encephalomyelitis (EAE) (Casey et al., 2022). Figure 1 outlines the roles of hepatic macrophages in liver inflammation.

## Hepatic macrophages as microbial regulating targets in inflammation-related liver diseases

The human gut contains numerous commensal and external micro-organisms that play important roles in host physiology (Garrett, 2017; Zmora et al., 2019). Microbial components and metabolites can translocate into distant organs, especially the liver, through the portal vein system (Cani, 2018; Albillos et al., 2020; Jones and Neish, 2020). Under conditions of eubiosis or dysbiosis, gut-derived microbial signals exert various effects on the liver, with hepatic macrophages acting as crucial players in the gut-liver axis (Bruneau et al., 2021; De Muynck et al., 2021).

Among the various microbial components that can stimulate hepatic macrophages, the most typical agent is probably LPS. Gut bacteria constitute an abundant pool of LPS, which continuously stimulates hepatic macrophages through the portal vein system. LPS/TLR4 signaling on KCs can sense endotoxin translocation and initiate the activation of various inflammatory responses (Janeway and Medzhitov, 2002; Krenkel and Tacke, 2017; Tripathi et al., 2018; Gu et al., 2021). Receptor interacting protein kinase 3 (RIP3) signaling, which is regulated by gut microbiota and endotoxin translocation, is also key to macrophage secretion proinflammatory agents (Zhang et al., 2020; Zhang et al., 2021b). Although physiologic doses of LPS are essential for the development of both immunity and tolerance (Chou et al., 2015; Zhou W. et al., 2022), excessive endotoxin translocation due to gut dysbiosis can induce the pathogenic activation of hepatic macrophages. Such aberrant activation is believed to induce deleterious inflammatory processes in many liver diseases, such as nonalcoholic fatty liver disease (NAFLD) (Liu et al., 2015; Wesolowski et al., 2017; Hegazy et al., 2020) and alcoholic liver disease (ALD) (Inokuchi et al., 2011; Szabo, 2015; Bajaj, 2019). In addition, the translocation of microbial DNA contributes to liver inflammation. Some subsets of hepatic macrophages are essential barriers to clear the enrichment of microbial DNA, whereas obesity can reduce and weaken this barrier (Luo et al., 2021; Luo et al., 2022).

Some microbial metabolites produced by probiotics also possess the ability to regulate the inflammatory activation of macrophages. Generally, metabolites with anti-inflammatory properties, such as SCFAs and indole derivatives, can improve gut dysbiosis and intestinal barriers, thus inhibiting endotoxin translocation-induced activation of hepatic macrophages (Bao et al., 2020; Wang Y. et al., 2021; Xia et al., 2022). Specifically, indole treatment can inhibit proinflammatory activation of hepatic macrophages in a manner dependent on 6-phosphofructo-2-kinase/fructose-2 and 6-biphosphatase3 (PFKFB3) (Ma et al., 2020). Treatment with SCFA was found to alter macrophage phagocytosis in other tissues, such as the lungs and brain, leading to the regulation of inflammation (Trompette et al., 2014; Erny et al., 2021).

The microbial interventions which can exert influence on hepatic macrophage and inflammation-associated liver diseases are concluded in Table 1. Subsequent sections of this review will concretely describe the crosstalk between gut microbiota and liver macrophages in various inflammation-related liver diseases. Findings have suggested that targeting hepatic macrophages may have therapeutic potential in the treatment of microbial diseases.

**TABLE 1:** Microbial intervention and their effects on hepatic macrophage and liver disease.

Microbial intervention	Effect on hepatic macrophage and liver disease	References
Prebiotics		
Inulin	Inhibits macrophages activated through HMGB-1/TLR4 signaling. Ameliorates IR induced-ALI with increased *Bacteroides acidifaciens* and short-chain fatty acids (SCFAs) especially propionic acid	Kawasoe et al. (2022)
	Reduces macrophage and its TLR4 signaling in liver. Ameliorates inflammation by hindering Nod-Like Receptor Protein 3 Inflammasome as well as NF-κB signaling in NAFLD.	Bao et al. (2020)
	Suppresses LPS-TLR4-Macrophage axis and induce M2 macrophage polarization. Alleviates inflammation of ALD *via* SCFAs	Wang et al. (2020b)
Fucoidan	Reduces macrophage infiltration in liver. Ameliorates liver inflammation in NAFLD	Li et al. (2018)
Fructo-oligo-saccharide	Decreases the abundance of KCs. Improves gut dysbiosis and inflammation in MCD-induced NAFLD	Matsumoto et al. (2017)
Probiotics		
*Akkermansia muciniphila*	Reduces macrophage infiltration in liver. Ameliorates inflammation in APAP-induced ALI.	Xia et al. (2022)
*Lactobacillus salivarius* Li01	Reduces macrophage infiltration in liver. Ameliorates inflammation in TAA-induced ALI.	Yang et al. (2020)
*Lactobacillus acidophilus* LA14	Represses macrophage inflammatory protein 1a (MIP-1a), macrophage inflammatory protein 3a (MIP-3a), and Monocyte chemoattractant protein-1 (MCP-1) levels. Improves d-galactosamine (D-GalN)-induces ALI.	Lv et al. (2021)
*Lactobacillus reuter*i GMNL-263	Inhibits MCP-1 level and macrophage infiltration in liver. Reverses HFD-induced metabolic impairment and liver inflammation	Hsieh et al. (2016)
*Lactobacillus paracasei*	Increase the fraction of M2 KCs. Alleviates liver inflammation in NAFLD mice	Sohn et al. (2015)
*Lactobacillus pentosus* strain S-PT84	Decreases macrophage infiltration and increase the fraction of M2 KCs in liver. Alleviates liver inflammation and fibrosis in NAFLD mice	Sakai et al. (2020)
*Lactobacillus rhamnosus* GG	Decreases the infiltration of hepatic macrophages. Leads to steatosis and inflammation alleviation in ALD.	Zhu et al. (2022b)
*Bifidobacterium animalis* ssp. Lactis 420	Modulates RIP3 signaling of hepatic macrophages. Alleviates S100-induced experimental autoimmune hepatitis (EAH)	Zhang et al. (2021b)
*Bifidobacterium pseudocatenulatum* CECT 7765	Modulates M1/M2 macrophages balance. Reduces obesity-associated liver inflammation	Moya-Perez et al. (2015)
*Clostridium butyricum* (plusing soluble dietary fiber)	Decreases the percentage of KCs and the activation of M1 macrophages in liver with inhibited TLR4/NF-κB signaling pathway. Alleviates liver inflammation in NAFLD mice	Shao et al. (2022)
*Pediococcus pentosaceus* CGMCC 7049	Decreases and monocytes/macrophages related MIP-1 and MCP-1. Improves SCFAs production and liver inflammation in ALD.	Jiang et al. (2020)
Microbial metabolites		
3,4-dihydroxy-phenylpropionic acid	Inhibits the pro-inflammatory activation of hepatic macrophages. Improves IR induced-ALI.	Li et al. (2022a)
Indole	Inhibits macrophage activation in a PFKFB3-dependent manner. Alleviates HFD or MCD-induced NAFLD severity	(Ma et al., 2020; Zhu et al., 2022a)
Indole-3-acetic acid	Promotes M2 macrophage differentiation. Relieves obesity-associated NAFLD.	Wang et al. (2021b)
Antibiotics	
Wide-spectrum antibiotic treatment (ABX)	Decreases infiltration of Ly6C^+^ monocytes and represses TLR signaling in KCs. Improve liver injury in ALI.	Kolodziejczyk et al. (2020)
	Inhibits macrophages infiltration in both liver and adipose tissue and related LPS and TLR4 signaling activation. Alleviates HFD-induced liver inflammation	(Carvalho et al., 2012; Yamada et al., 2017)
	Induces the production of IL-10 from KCs with impaired gut microbiota. Leads to T cell suppression which hinders HBV clearance	Zhou et al. (2022b)
Amphotericin B	Inhibits *β*-glucan translocation-activated C-type lectin–like receptor (CLEC7A) on KCs. Ameliorates ALD inflammation	Yang et al. (2017)

### Acute liver injury

Acute liver injury (ALI) is a clinical syndrome of multiple causes characterized by a massive inflammatory response and large-scale hepatocyte necrosis (European Association for the Study of the Liver et al., 2017; Stravitz and Lee, 2019). In experimental models of ALI, hepatic macrophages generally share common roles in disease development. By sensing injury, releasing cytokines, and infiltrating liver tissue, hepatic macrophages play vital roles in the exacerbation or resolution of inflammation (Tacke, 2017; Li W. et al., 2022).

Both the gut microbiome and hepatic macrophages were shown to be important in ALI. Disease phenotypes of drug-, chemical-, and endotoxin-induced ALI were found to be milder in the presence than in the absence of wide-spectrum antibiotic treatment (ABX), as well as being milder in germ-free (GF) than in non-GF mice. The lack of endogenous microbial stimulation was found to ameliorate liver inflammation and subsequent tissue injury (Lin et al., 2012; Kolodziejczyk et al., 2020; Zheng et al., 2022). Single cell transcriptome analysis showed that, compared with mice having intact microbiomes, mice lacking microbiomes exhibited significantly reduced infiltration of Ly6C^+^ monocytes, perhaps due to repressed TLR signaling in KCs (Kolodziejczyk et al., 2020). Translocation of both DAMPs and microbe-associated molecular patterns (MAMPs) in a reperfusion/ischemic (IR) intestine model was found to trigger KC polarization to an M1 phenotype, leading to hepatic inflammation *via* the HMGB1/TLR4/RAGE axis (Wen et al., 2020). The common commensal bacteria *Bifidobacterium longum* and *B. fragilis* were shown to induce secretion of the proinflammatory cytokine IL-1b from human THP-1 macrophages *in vitro*, suggesting possible gut microbiome-macrophage-ALI interactions (Shanmugam et al., 2015). Translocation of all gut-derived MAMPs, except for *in situ* DAMPs resulting from direct damage to hepatocytes, was also found to exacerbate ALI *via* hepatic macrophages.

Conversely, microbial intervention has been shown to reduce liver inflammation by reducing the activity of hepatic macrophages in animal models of ALI. Pretreatment of mice with acetaminophen (APAP)-induced ALI with the probiotic, *Akkermansia muciniphila*, was found to improve gut leakage and decrease the infiltration of hepatic macrophages, as shown immunohistochemically by lower levels of the monocyte/macrophage markers F4/80 and Ly6G, followed by reductions in the concentrations of macrophage-related proinflammatory cytokines (Xia et al., 2022). *Lactobacillus salivarius* Li01 also had a similar effect on mice with thioacetamide (TAA)-induced ALI (Yang et al., 2020). Moreover, *L. acidophilus* LA14 was found to reduce d-galactosamine (D-GalN)-induced ALI by lowering the concentrations of macrophage inflammatory protein 1a (MIP-1a), MIP-3a, and monocyte chemoattractant protein-1 (MCP-1), suggesting reductions in macrophage activity (Lv et al., 2021). Except for toxin-induced ALI, *Bifidobacterium animalis* ssp. Lactis 420 (*B. 420*) was found to alleviate S100-induced experimental autoimmune hepatitis (EAH) by modulating RIP3 signaling of hepatic macrophages (Zhang et al., 2020). In addition, an inulin diet was shown to ameliorate IR-induced ALI, accompanied by significant increases in the abundance of *Bacteroides acidifaciens* in feces and its SCFAs, especially propionic acid (PA), in the portal vein (Kawasoe et al., 2022). Furthermore, PA alone was sufficient to inhibit macrophages activated through HMGB-1/TLR4 signaling *in vitro* (Kawasoe et al., 2022). The gut microbiota metabolite 3,4-dihydroxyphenylpropionic acid was shown to play a similar role in IR-induced ALI (Li R. et al., 2022).

Macrophage-based cell therapy has been shown to be effective to induce the resolution of inflammation. For example, administration to mice of *in vitro*-induced M2 macrophages, mainly Lyc6^-^ macrophages, significantly alleviated liver inflammation and stimulated hepatocyte proliferation, even after hepatic necrosis had been established. A similar effect was observed when human-derived M2 macrophages were administered to immunocompetent mice, suggesting that similar treatment may have promising potential in clinical practice (Starkey Lewis et al., 2020). Meanwhile, recent studies indicated the capability of microbial intervention to regulate macrophage polarization. Both pathogenic bacteria that facilitate tumorigenesis and beneficial bacteria that regulate inflammation were capable of inducing M2 macrophage polarization (Li et al., 2019; Wang Y. et al., 2020; Wang Z. et al., 2020; Xu et al., 2021). Additional studies, however, are needed to identify the underlying mechanisms of action of microbial intervention in the treatment of ALI.

### Nonalcoholic fatty liver disease

Changes in lifestyle and dietary habits have increased the numbers of patients with nutrient/metabolism problems such as NAFLD (Younossi et al., 2018). NAFLD is a chronic process, starting from incipient steatosis and extending to advanced steatohepatitis, which is characterized by subacute liver inflammation (Schuster et al., 2018). Hepatic macrophages play a crucial role in impairing the metabolism of fatty acids and in initiating fibrogenesis (Tacke, 2017; Friedman et al., 2018), with the gut-liver axis being an important contributor to the development of NAFLD (Wesolowski et al., 2017; Friedman et al., 2018).

Administration of a high-fat diet (HFD) is a common experimental treatment to induce insulin resistance and subclinical inflammation, which are fundamental characteristics in NAFLD development. ABX treatment can alleviate HFD-induced inflammation, accompanied by significant reductions in macrophage infiltration into both liver and adipose tissue. Reductions in LPS and TLR4 signaling activation are accompanied by improved insulin resistance (Carvalho et al., 2012; Yamada et al., 2017). HFD was shown to reduce the numbers of Vsig4^+^ KCs, impairing the ability to clear microbiota-derived extracellular vesicles (EVs) (Luo et al., 2021). Marked accumulation of bacterial DNA has been observed in the livers of Vsig4^−/−^ mice, with this DNA being responsible for liver inflammation and the fibrogenic activation of hepatic stellate cells (HSCs) (Luo et al., 2022). Biopsies of liver tissue from 60 patients with NAFLD and 40 healthy controls showed that the levels of the KC activation markers LPS and CD 163 were higher in the former (Hegazy et al., 2020). The increases in LPS and KC activation, along with NAFLD progression, confirmed the involvement of gut leakage and the microbiome-macrophage axis in the development of NAFLD (Hegazy et al., 2020). In addition, the microbial byproducts, volatile organic compounds (VOCs), were found to translocate into the liver through the portal vein system in methionine- and choline-deficient (MCD) diet-induced NAFLD mice (Reid et al., 2016). These VOCs were toxic to hepatic macrophages and induced further release of proinflammatory cytokines (Reid et al., 2016). A study of 34 patients with NAFLD found a negative correlation between the bacterium *Faecalibacterium prausnitzii* and cells positive for CD163^+^, a surface marker of KCs in the portal tract (r = −0.371; *p* = 0.022) (Schwenger et al., 2018). Taken together, these findings indicated that the crosstalk between gut-derived microbial components and hepatic macrophages was crucial in the development of NAFLD/NASH.

The abilities of anti-inflammatory agents and macrophages to regulate microbial intervention have been established. For example, administration of either heat-killed (HK) or live *Lactobacillus reuteri* GMNL-263 (*Lr*263) was found to reverse HFD-induced metabolic impairment and liver inflammation, as well as reducing MCP-1 levels and macrophage infiltration in the liver (Hsieh et al., 2016). Dietary fructo-oligosaccharide, acting as a prebiotic, was found helpful in restoring intestinal barriers and gut eubiosis, reducing inflammation and KC populations (Matsumoto et al., 2017). Similar effects were observed when other prebiotics and functional foods, including inulin (Bao et al., 2020), fucoidan from the sea cucumber *Pearsonothuria graeffei* (Li et al., 2018), and *Sarcodon aspratus* polysaccharides (Chen et al., 2020), were administered to HFD mice. These findings suggest that the therapeutic potential of prebiotics or functional foods in NAFLD was due partly to their effects on the microbiome-hepatic macrophage axis. The microbial metabolite indole was shown to alleviate the severity of HFD- or MCD-induced NAFLD by inhibiting macrophage activation in a PFKFB3-dependent manner (Ma et al., 2020; Zhu B. et al., 2022). Moreover, a study using a GF model found that transplantation of fecal microbiota from 2-week-old infants born to obese (Inf-ObMB) mice to GF mice resulted in low-grade inflammation and susceptibility to NAFLD. Moreover, the functions of hepatic macrophages from Inf-ObMB mice were impaired, as shown by damaged gut barrier, macrophage phagocytosis, and cytokine production. These findings indicate that the microbiome-hepatic macrophage axis acts as a potent player in maternal obesity-associated childhood obesity and NAFLD (Soderborg et al., 2018).

The balance of M1/M2 macrophages in the liver is a promising target for microbial intervention in NAFLD progression (Murray, 2017; Wang C. et al., 2021). Supplementation with *Bifidobacterium pseudocatenulatum* CECT 7765 can reduce obesity-associated inflammation by modulating the balance of M1/M2 macrophages and increasing the Treg population (Moya-Perez et al., 2015). In addition, *Lactobacillus paracasei* and *L. pentosus* strain S-PT84 were found to improve gut dysbiosis, increase the fraction of M2 macrophages in NAFLD mice, and alleviate inflammation response (Sohn et al., 2015; Sakai et al., 2020). The microbial metabolite, indole-3-acetic acid (I3A), which is elevated after sleeve gastrostomy, was found to promote M2 differentiation both *in vivo* and *in vitro*, relieving obesity-associated NAFLD (Wang Y. et al., 2021). Further, coadministration of *Clostridium butyricum* and soluble dietary fiber reduced the percentage of KCs and the activation of M1 macrophages in liver by suppressing the TLR4/NF-κB signaling (Shao et al., 2022).

In addition to their anti-inflammatory activities, hepatic macrophages have autophagic activity, which might affect NAFLD. For example, an HFD was found sufficient to induce the impairment of macrophage autophagy, skewing macrophage polarization to the M1 subset (Liu et al., 2015). In contrast, administration of M2 macrophages was found to improve autophagic activity during liver inflammation (Starkey Lewis et al., 2020). This interaction between macrophage polarization and autophagy may be a target for long-term microbial intervention.

### Alcoholic liver disease

ALD may be the most prevalent chronic liver disease in the world (Rehm et al., 2013; Seitz et al., 2018). Hepatic macrophages have been shown to be important in the progression of ALD from hepatic steatosis to steatohepatitis (Krenkel and Tacke, 2017; Tacke, 2017). In addition to the direct injury to hepatocytes caused by ethanol metabolism, the close relationships among ethanol, the gut-liver axis, and ALD have raised increasing concerns. Alcohol-induced gut leakage and dysbiosis, along with the long-term stimulation of bacterial translocation, may activate hepatic macrophages, thereby contributing to the progression of ALD (Szabo, 2015; Tacke, 2017; Bajaj, 2019). However, Duan et al. found that CRIg + macrophages in liver, a novel subset of KCs, was reduced in patients with chronic alcohol assumption. Further study indicated that CRIg + macrophages can protect mice from ethanol-induced liver injury probably due to its ability to prevent bacteria translocation (Duan et al., 2021). Dysbiosis has been widely verified in patients with ALD, as illustrated in a recent comprehensive review (Bajaj, 2019). Generally, ALD patients exhibit some common traits such as a reduced abundance of Bacteroidetes, an increased abundance of bifidobacterial, and a correlation between increasing endotoxemia and disease progression (Mutlu et al., 2012; Llopis et al., 2016; Dubinkina et al., 2017; Ciocan et al., 2018).

Microbial intervention targeting the intestinal barrier may have therapeutic potential in patients with ALD (Grander et al., 2018; Seo et al., 2020; Li et al., 2021), as may microbial intervention targeting the microbiome-hepatic macrophage axis. For example, supplementation with *Lactobacillus rhamnosus* GG (*LGG*) was found to significantly reduce the infiltration of hepatic macrophages, alleviating steatosis and inflammation (Zhu Y. et al., 2022). *Pediococcus pentosaceus* CGMCC 7049 was found to reduce liver inflammation and monocyte/macrophage secretion of MIP-1 and MCP-1 by reversing ethanol-induced gut dysbiosis and enhancing the production of SCFAs (Jiang et al., 2020). Gut mycobiota also show crosstalk with hepatic macrophages in ALD. For example, chronic alcohol administration was found to lead to intestinal fungal overgrowth and *β*-glucan translocation in mice. *β*-Glucan exacerbates liver inflammation through the C-type lectin–like receptor (CLEC7A) on KCs. Treatment with the antifungal agent amphotericin B can block this signaling and ameliorate ALD-associated inflammation (Yang et al., 2017).

In contrast to these findings, a recent clinical study found that, compared with standard of care, treatment of 31 ALD patients with ABX for 7 days had no effect on LPS-binding protein (LBP), markers of hepatic macrophage activation, and systemic inflammation levels (Støy et al., 2021). Such contradiction indicated that gut leakage and the gut-microbiota-hepatic macrophage axis play a reduced role in patients with established ALD, but further studies are necessary to determine the role of gut leakage in ALD.

### Viral hepatitis

The chronicity of HBV infection is a major clinical concern, and is thought to involve a balance between immune tolerance and activation. The microbiome-hepatic macrophage axis is thought to play a role in virus clearance and tolerance. In contrast to normal development from immune tolerance to immune activation with age, ABX treatment was found to inhibit HBV clearance in adult mice, although TLR4-mutant young mice exhibited rapid HBV clearance (Chou et al., 2015). These findings suggested that the stability and maturity of gut microbiome-TLR4 signaling in KCs play an important role in HBV immunity (Chou et al., 2015). ABX-induced dysbiosis was found to induce KC secretion of IL-10, which leads to T cell suppression, inhibiting HBV clearance and facilitating the chronicity of HBV infection (Zhou W. et al., 2022). Taken together, these studies indicate that physiologic stimulation with components of gut-derived microbes such as LPS is essential to the development and maintenance of activated KCs against HBV infection. In addition, a subset of intrahepatic myeloid cells, CD14^+^HLA^−^DR^hi^CD206^+^ macrophages, activated by translocated microbial components can promote liver inflammation in chronic virus-associated liver diseases (Tan-Garcia et al., 2017).

## Summary

**FIGURE 2:**
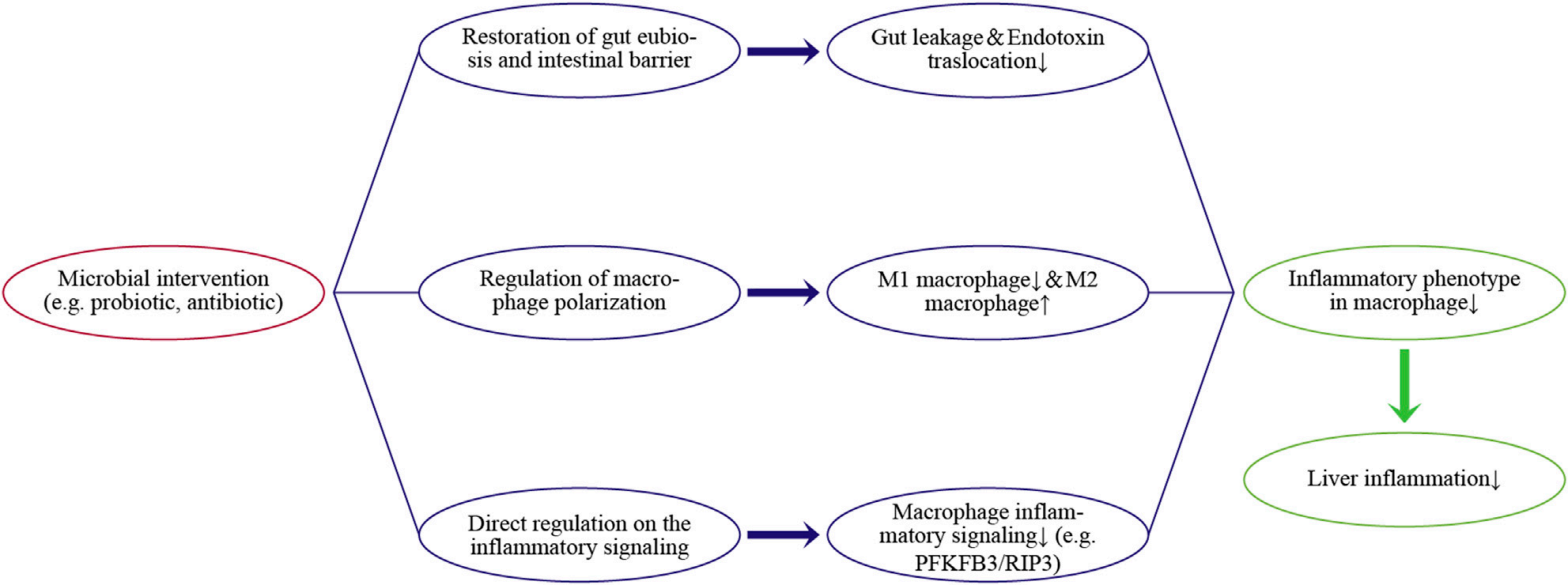
Regulating pathways of the microbial intervention on the inflammatory phenotype in hepatic macrophages and liver inflammation.

Evaluation of the gut-liver axis has shown that inflammatory responses in the liver are a common but troublesome health problem due to various external chemical agents, such as alcohol, lipids, and drugs, as well as invasion by gut micro-organisms. Hepatic macrophages are a key player in liver homeostasis and responses to inflammation, as well as having close relationships with gut microbiota. Hepatic macrophages can regulate gut-derived inflammatory signals and maintain immune tolerance under physiologic conditions. However, disruption of the intestinal barrier due to dysbiosis aggravates the translocation from gut-derived inflammatory signals. This aberrant exposure induces an excessive proinflammatory phenotype in macrophages, thereby contributing to inflammation-related liver diseases. Thus, microbial interventions, such as reducing bacteria load with antibiotics or improving intestinal barrier with pre/probiotics, can indirectly alleviate the activation and infiltration of hepatic macrophages. Nevertheless, the clinical ability of microbial interventions to regulate endotoxin translocation remains unclear in some cases. Additional clinical trials of microbial interventions in patients with established liver inflammation are needed. In addition, several microbial metabolites have exhibited the potential to directly suppress inflammatory signaling or induce macrophage polarization toward the anti-inflammatory phenotype (e.g., M2 cells). Regulation of the inflammatory phenotype of macrophages is a potent target for microbial intervention, especially in chronic inflammatory responses. Figure 2 concludes the main pathways.

In conclusion, recent studies have indicated the importance of gut microbiota signals on the inflammatory phenotype in hepatic macrophages. Crosstalk between hepatic macrophages and gut microbiota is a promising target to regulate inflammatory responses and alleviate related liver diseases.
